# Technological advancement in the era of COVID-19

**DOI:** 10.1177/20503121211000912

**Published:** 2021-03-09

**Authors:** Nishant Renu

**Affiliations:** Doctor of Business Administration, Westcliff University, Irvine, CA, USA

**Keywords:** COVID 19, Internet, robots, technology, telehealth

## Abstract

Regional and local governments worldwide are working tirelessly toward effective ways of addressing the COVID-19 crisis. During this time, the government has had to ensure that they provide full usage of technological means to confront the pandemic and discourse a wide range of COVID-19 related problems. Herein, this article will discuss the application of technical means and the advancement of technology in different sectors as a consequence of the COVID-19 crisis. Further, it highlights how government and health organizations have introduced new policies intending to try to curb the spread of the coronavirus. These new policies, such as lockdowns and social distancing measures, have resulted in technological advancement and new means of interaction with government, businesses, and citizens. Such changes include increased online shopping, as well as robotic delivery systems, the introduction of digital as well as contactless payment systems, remote working, the role of technology in distance learning, Telehealth, 3D Printing, and online entertainment. These technological advancements have been embraced all the way during this pandemic by a few countries around the world, with its limitation in some underdeveloped and developing countries.

## Introduction

Coronavirus pandemic has posed a significant impact on an individual’s life, both negative and positive. Due to the increase of the coronavirus pandemic at an alarming rate globally, every individual has to revisit the global norms. The global norms have usually been accepted to solve complex development challenges on the ground and are deemed crucial for societies to flourish. Therefore, to change the entire geopolitical systems, the government has endorsed new methods of applying technology to positively impact the community and encourage ongoing activities for every individual. Due to the coronavirus pandemic, there has been a significant effect on the running of the economy by the government by introducing new methods of technology to ensure activities are ongoing as well as they are done more effectively.^1^ Typically, government information has focused on addressing the public by giving out detailed information about the outbreak of the disease and imposing strategies and policies to be followed, such as restrictions on traveling and social distancing among individuals, hence assessing technology advancement.^2^ The discussion of the technological progress will concentrate mainly on virtual learning among students and scholars, online purchasing of merchandise, the use of the robotic delivery system, implementation of online entertainment, and contactless payments through cards and e-wallets.

Therefore, this article is tailored to explore the advancement of technology in this era of COVID-19 and how it has impacted individuals’ lives and states in a few developed and technologically advanced countries.

## Technologies have improved online shopping and robotic deliveries

**Figure 1.**
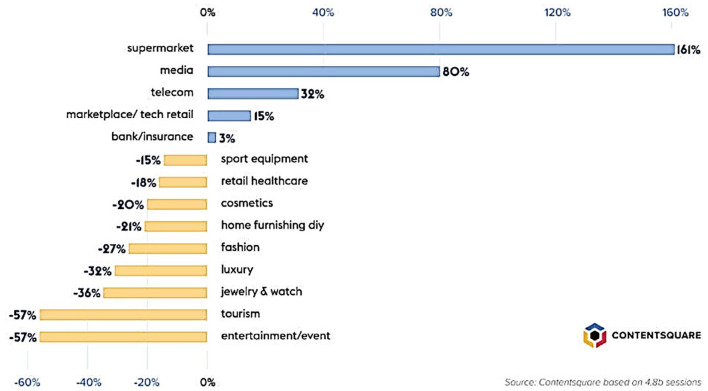
Coronavirus impact on online traffic by industry by Jean-Marc Bellaiche, 2020. Retrieved from https://contentsquare.com/blog/impact-of-coronavirus-on-ecommerce-consumers-settle-into-quarantine. Copyright 2020 by Contentsquare. Reprinted with permission.

After the outbreak of COVID- 19 in the world, technological advancement has been used to promote and enable the business to continue running throughout. COVID-19 has transformed online shopping from occasional to a must globally to minimize the movement of people all over, thus controlling the coronavirus’s spread.^3^ Online shopping is enhanced through robust logistics systems where robots are being used as the means to deliver food supplies and other commodities because in-person delivery isn’t virus-proof.^4^ Countries like China and the United States have launched contactless delivery services where the customer’s goods ordered are élite and dropped off at the selected locations instead of the customers picking for themselves using their hands. However, not every sector is equal in terms of e-commerce amid this pandemic. Significant variations and changes in online behavior have been observed (Figure 1).^5^ There is a considerable rise in traffic in some sectors, while others are seeing a significant decrease in digital visits.

## Technology has accelerated digital and contactless payments

Coronavirus is considered a contagious disease. The virus can stay on the surfaces for more than 24 hours; thus, payment through cash is discouraged to prevent the spread of coronavirus to those who are not infected.^6^ As a result of advancements in technology, different countries have employed the use of soft money or contactless payments to pay for any services. Several banks in China, the United States, and South Korea have instigated various methods to ensure banknotes are uncontaminated before circulating in the market. Different platforms have been designed to allow online purchases and marketing of goods and services effectively without the physical entanglement of customers.^7^ In addition, it has made utility payments and allocation of stimulus funds faster due to the availability of high-speed Internet and improved devices, which are relatively quicker in transferring cash in a digitalized format.

## Remote working

Technology has made working remotely more effective. Since the outbreak of the COVID-19 pandemic globally, many companies and business organizations have pleaded their workers to work from home to prevent direct contact and mingling with other work staff.^8^ Technology facilitates remote working through the use of virtual private networks, voice over Internet protocols, enabling virtual meeting through zooms or google platforms, and with the use of facial recognition technologies allowing the person to appear behind the virtual background. In addition to preventing the coronavirus spread, remote working has helped save several unnecessary meeting hours and providing flexibility to the business employees. Although remote working is enhanced by technology, it imposes a lot of challenges to employers and employees.^9^ The security of information and privacy is a big concern while working virtually. So, laws and regulations must be put in place to prevent such issues. It can also complicate the labor laws where the companies may tend to hire people from areas with cheaper labor costs. The government should look into these challenges on an individual basis to avoid any complications in the near future.

## Distance learning

Technology has improved distance learning among the students and their teachers. Due to the increasing number of patients infected with the coronavirus, many countries issued the cessation of all in-person learning classes in institutes to help thwart the coronavirus spread.^10^ Many institutions started offering online classes through online platforms such as Google or Zoom to ensure that the quarantine measures didn’t disrupt education.^11^ Technology implemented in distance learning is the same used to enhance effective remote working. This new online technology also involves the use of artificial intelligence-enabled robotic teachers. Students engaged in distance learning are getting skilled in several Internet-based technologies, making them think critically and become innovative.

## Telehealth

Advanced technology has improved Telehealth and the administration of healthcare to patients with the coronavirus. Telehealth is an effective way made possible by technology to prevent the spread of COVID 19 and provide essential primary care to patients.^12^ The doctors can diagnose patients through the description they give via the chat box or video conferencing using their cell phone, computer, or laptop, and offer guidelines in real time on what is to be done or prevented. Telehealth adoption by the healthcare administrations is readily bridging the gap between physicians, patients, and health systems, enabling everyone, symptomatic patients especially, to interact through virtual channels with their doctors from the comfort of their home, further helping in reducing the spread of the virus to mass populations and the medical staff on the frontlines.^13^ Nowadays, all healthcare practitioners have remote devices set up for patient monitoring and online care. These remote networks are actively capturing and submitting data for interpretation to other healthcare organizations. This is an important move in telemedicine when you can quickly get the new health updates to your doctor even though you are homebound. Depending on how your telehealth program is set up with the healthcare provider, you can modify, change, and use the telemedicine service for your consultation. Your healthcare provider can forward diagnostic images to the telemedicine doctor for them to examine, such as X-rays and your medical records. The telemedicine practitioner can make a diagnosis and also establish an effective recovery plan online after reviewing the medical records. They can also forward your prescription to your nearest pharmacy online and get it delivered to your door steps.^14^ The usage of artificial intelligence and robotic-assisted telemedicine has several potential applications in performing a patient diagnosis, monitoring, and clinical care in remote areas. Robotic-assisted telemedicine has played a significant role in providing support to the patients infected with the coronavirus and avoid direct contact with the doctors.^15^ Nonetheless, robots are also being used to disinfect areas and surfaces where the virus is suspected to be significantly affected or on the public services areas where lots of people use or gather.^16^ They have also been used for food deliveries to families during the quarantine in certain countries.^17^ Drones have been used to monitor people and deliver items needed in hospitals to take care of patients.^18^ Considering these advancements, many people suggest that robots will limit human interactions to a greater extent and replace manufacturing jobs in the future. At the same time, it will lead to the creation of new positions in the tech industry.

## Online entertainment

Online entertainment has been enhanced by technology tremendously during this time. Although personal interactions have been reduced by the quarantine measures placed to prevent the spread of coronavirus, different ways have been innovated to bring parties online. All over the world, different platforms have been created to bring the music and entertainment industries together. Cloud raves and online streaming are significant ways where many people tend to get unmaintained through listening to musicians or actors of their choice all over the world.^19^ The outbreak of COVID 19 resulted in the cancelation of many movements and any forms of gatherings that enabled the museum’s and international heritages site to offer virtual tours the entire world. Many people have started embracing online games since the outbreak to keep them engaged and entertained. Digital streaming and binge-watching have become a regular phenomenon for others looking for online entertainment.

## 3D printing

Government organizations, as well as private organizations, have introduced the 3D printing technology to ensure mitigation of shocks from the exporting bans that play the responsibility of personal protective equipment. 3D printing is useful in building mockups and producing various items based on different materials and designs. This helps make simple parts easily assembled onsite, even without requiring a full procurement.^20^ Recently, 3D printing technology was used to design surgical masks for the doctors involved in operating the individuals who have been affected by COVID-19 disease. These 3D printed masks have been really effective in reducing virus exposure and may be worn as an alternative to surgical type masks.^21^ Therefore, it is evident that the advancement of technology on 3D printing has created a positive impact by driving creativity and innovation.

## Limitations of this study

As with the majority of studies, the design of the current study is subject to certain limitations. The primary limitation of this review is that its tailored to highlight some of the technological advancements that took place only in a few developed and certain developing countries while dealing with the current COVID-19 pandemic. More in-depth study and research is needed to identify measures and technological advancements that some other least developed and developing countries are taking to overcome the challenges associated with this unprecedented time.

## Conclusion

The rise of the Coronavirus disease has gradually led to changes in individuals’ lives in both positive and negative ways. Equitable access to the application of various digital infrastructures has been considered to be essential right now. The demand for advancement in technology is to respond to the current implications of COVID-19 disease. It is clear that as far as concerned, the rapid application of the new technological methods to curb the current emergency has posed a broad and wide digital division. Even if the digital divide’s existence is not new, the present disaster has added a new dimension of addressing urgent issues. Through the application of policies imposed by the government and the world health organization toward social distancing, maintenance of basic hygiene and traveling restrictions has taught individuals to be responsible for their own health and how to respond to urgent issues when they arise. Besides, the advancement of technology has played its best role in ensuring and maintaining ongoing activities without interruption. Therefore, it is crystal clear through the past discussion above that technological advancement during this era of COVID-19 has significantly impacted individuals’ activities and states in a few countries.
